# In-Home Coal and Wood Use and Lung Cancer Risk: A Pooled Analysis of the International Lung Cancer Consortium

**DOI:** 10.1289/ehp.1002217

**Published:** 2010-09-15

**Authors:** H. Dean Hosgood, Paolo Boffetta, Sander Greenland, Yuan-Chin Amy Lee, John McLaughlin, Adeline Seow, Eric J. Duell, Angeline S. Andrew, David Zaridze, Neonila Szeszenia-Dabrowska, Peter Rudnai, Jolanta Lissowska, Eleonóra Fabiánová, Dana Mates, Vladimir Bencko, Lenka Foretova, Vladimir Janout, Hal Morgenstern, Nathaniel Rothman, Rayjean J. Hung, Paul Brennan, Qing Lan

**Affiliations:** 1 Division of Cancer Epidemiology and Genetics, National Cancer Institute, Bethesda, Maryland, USA;; 2 International Agency for Research on Cancer, Lyon, France;; 3 Tisch Cancer Institute, Mount Sinai School of Medicine, New York, New York, USA;; 4 International Prevention Research Institute, Lyon, France;; 5 Department of Epidemiology, University of California School of Public Health, Los Angeles, California, USA;; 6 Samuel Lunenfeld Research Institute, Toronto, Ontario, Canada;; 7 National University of Singapore, Singapore;; 8 Unit of Nutrition, Environment and Cancer, Cancer Epidemiology Research Programme, Catalan Institute of Oncology, Barcelona, Spain;; 9 Norris Cotton Cancer Center, and Department of Community and Family Medicine, Dartmouth Medical School, Lebanon, New Hampshire, USA;; 10 Institute of Carcinogenesis, Cancer Research Center, Moscow, Russia;; 11 Department of Epidemiology, Nofer Institute of Occupational Medicine, Lodz, Poland;; 12 National Institute of Environmental Health, Budapest, Hungary;; 13 Cancer Center and Maria Sklodowska-Curie Institute of Oncology, Warsaw, Poland;; 14 Department of Occupational Health, Specialized State Health Institute, Banska Bystrica, Slovakia;; 15 Institute of Hygiene, Public Health, Health Services, and Management, Bucharest, Romania;; 16 Institute of Hygiene and Epidemiology, First Faculty of Medicine, Charles University, Prague, Czech Republic;; 17 Department of Cancer Epidemiology and Genetics, Masaryk Cancer Institute, Brno, Czech Republic;; 18 Department of Preventive Medicine, Faculty of Medicine, Palacky University, Olomouc, Czech Republic;; 19 Departments of Epidemiology and Environmental Health Sciences, School of Public Health, and Comprehensive Cancer Center, University of Michigan, Ann Arbor, Michigan, USA

**Keywords:** coal, lung cancer, pooled, risk factor, wood

## Abstract

**Background:**

Domestic fuel combustion from cooking and heating is an important public health issue because roughly 3 billion people are exposed worldwide. Recently, the International Agency for Research on Cancer classified indoor emissions from household coal combustion as a human carcinogen (group 1) and from biomass fuel (primarily wood) as a probable human carcinogen (group 2A).

**Objectives:**

We pooled seven studies from the International Lung Cancer Consortium (5,105 cases and 6,535 controls) to provide further epidemiological evaluation of the association between in-home solid-fuel use, particularly wood, and lung cancer risk.

**Methods:**

Using questionnaire data, we classified subjects as predominant solid-fuel users (e.g., coal, wood) or nonsolid-fuel users (e.g., oil, gas, electricity). Unconditional logistic regression was used to estimate the odds ratios (ORs) and to compute 95% confidence intervals (CIs), adjusting for age, sex, education, smoking status, race/ethnicity, and study center.

**Results:**

Compared with nonsolid-fuel users, predominant coal users (OR = 1.64; 95% CI, 1.49–1.81), particularly coal users in Asia (OR = 4.93; 95% CI, 3.73–6.52), and predominant wood users in North American and European countries (OR = 1.21; 95% CI, 1.06–1.38) experienced higher risk of lung cancer. The results were similar in never-smoking women and other subgroups.

**Conclusions:**

Our results are consistent with previous observations pertaining to in-home coal use and lung cancer risk, support the hypothesis of a carcinogenic potential of in-home wood use, and point to the need for more detailed study of factors affecting these associations.

Globally, lung cancer is estimated to account for almost 1.4 million incident cases of cancer each year and has been the most common cancer in the world for more than two decades (Parkin et al. 2005). Smoke from domestic fuel (i.e., coal, wood, biomass) used for cooking and heating has been associated with a variety of health outcomes (Kim and Hanley 2002; Kiraz et al. 2003; Mishra et al. 1999, 2004; Peters et al. 1999; Pintos et al. 1998; Pokhrel et al. 2005; Schei et al. 2004; Shrestha and Shrestha 2005; Tang et al. 2006; Wichmann and Voyi 2006), including lung cancer (Hernández-Garduño et al. 2004; Hosgood et al. 2008; Lan et al. 2002, 2008; Mumford et al. 1987). The annual global health burden of indoor air pollution from solid-fuel use, to which 3 billion people are exposed worldwide, is estimated to be 1.6 million deaths and > 38.5 million disability-adjusted life years (Ezzati and World Health Organization 2004; World Resources Institute et al. 1996).

The type of solid fuel used varies by region, with China using mostly coal and Western countries using wood. Throughout Asia, coal combustion for heating and cooking increases the levels in the home of known carcinogens such as polycyclic aromatic hydrocarbons (PAHs) [International Agency for Research on Cancer (IARC) 1983; Zhang and Smith 2003]. In Western countries, the use of wood-burning stoves in homes has been found to elevate levels of carcinogenic agents such as PAHs, benzene, and 1,3-butadiene (Gustafson et al. 2007, 2008).

Recently, IARC (2010) concluded that indoor emissions from household combustion of coal are carcinogenic to humans (group 1) and that indoor emissions from biomass, primarily wood, were classified as probable human carcinogens (group 2A). To further elucidate the association between lung cancer and solid-fuel use, particularly wood, we conducted a pooled analysis of seven epidemiologic studies with data on fuel use that evaluated this association and were included in the International Lung Cancer Consortium (ILCCO).

## Materials and Methods

Data from seven case–control studies involved in ILCCO, in which data on solid-fuel use were collected, were pooled for this analysis (Table 1). All seven studies have been previously described (Hashibe et al. 2006; Heck et al. 2009; Hung et al. 2008; Lan et al. 2000, 2008; Scélo et al. 2004; Seow et al. 2000). Overall, three studies were from North America, three from Asia, and one from Europe (Table 1). Population-based controls were enrolled in four studies, hospital-based controls were enrolled in two studies, and a mixture of both population- and hospital-based controls were enrolled in one study. Cases and controls were matched for at least age and sex in all studies; some studies matched for additional factors, such as local village. Informed written consent was obtained from all participants, and each study had the appropriate ethical approval from their respective institutions.

In total, 11,689 subjects (5,117 cases, 6,572 controls) were available for this analysis; of these, 35 participants were excluded because of missing education data, and an additional 14 were excluded because of missing smoking data.

Questionnaire data for the remaining 11,640 individuals were evaluated for in-home fuel exposures. Subjects were questioned about their use of fuel for heating and cooking throughout various points of their lives in six of the studies. The New England and California studies provided information on the main heating and cooking fuel for individuals during both childhood and adulthood. The Toronto, Central and Eastern Europe (CEE), and two Xuanwei studies provided information on the main heating and cooking fuel for multiple homes throughout the lifetime of the participants. The Singapore study provided the frequency of each fuel type used for cooking. For all individuals, the predominant fuel type, including electric, oil, gas, coal, and wood, that was used throughout their lifetimes was determined by a specific question in the questionnaire that asked which of all the fuel types was the predominant fuel used (Toronto), which was the fuel used as an adult (CEE, New England, California), which fuel was used for the longest period of time based on years of occupancy in each home (Xuanwei1, Xuanwei2), or which fuel was used most frequently for cooking (Singapore). The goal was to categorize subjects by the specific fuel type they used for the greatest number of years in their lifetimes. Each study developed their own questionnaire; details of these surveys and the methods used to administer them have been reported elsewhere (Hashibe et al. 2006; Heck et al. 2009; Hung et al. 2008; Lan et al. 2000, 2008; Scélo et al. 2004; Seow et al. 2000).

We classified the fuel type used throughout a lifetime into predominant solid-fuel users (coal, wood: 3,557 cases and 3,803 controls) and nonsolid-fuel users (electric, oil, gas: 1,548 cases and 2,732 controls). Further classification by specific fuel type identified 3,888 predominant coal users and 2,252 predominant wood users. The other 1,220 predominant solid-fuel users could not be classified as either predominant coal or predominant wood users because they consistently used both fuel types throughout their lives. Because nonsolid fuels produce substantially less smoke and are associated with fewer adverse health effects than are solid fuels (Haines et al. 2007), such as coal and wood, we considered nonsolid-fuel users to be the unexposed subjects for this analysis. Finally, individuals who used the same fuel source throughout their entire lives were classified as lifetime users: 1,818 lifetime solid-fuel users (939 cases, 879 controls), of which 1,267 were lifetime coal users (711 cases, 556 controls) and 218 were lifetime wood users (93 cases, 125 controls). We could not classify the other 333 lifetime solid-fuel users as either lifetime coal or lifetime wood users because they used both fuel types throughout their lives.

The lung cancer risk [odds ratios (ORs) and 95% confidence intervals (CIs)] associated with each fuel type was calculated by unconditional logistic regression, using SAS 9.1 (SAS Institute Inc., Cary, NC, USA). ORs and 95% CIs were adjusted for age (≤60, > 60 years), sex, education (low, medium/high), race/ethnicity (white, Asian, other), study center, and smoking status (ever, never). Because the covariates had different effects across the studies that evaluated wood use and lung cancer association, we included product terms between the covariates and studies in these pooled analyses. When ORs and 95% CIs were adjusted for more refined variables for age (≤50, 50–60, 60–70, > 70 years) and smoking status (never, ex-smoker, current smoker) we obtained similar results. In the subset of subjects where data were available, ORs and 95% CIs that were adjusted for pack-years smoked also yielded similar results. We also calculated ORs and 95% CIs by sex, smoking status, race/ethnicity, and geographic location. The heterogeneity across studies was evaluated by comparing the log-likelihood ratios of the logistic regression models with and without the product of fuel use and study.

## Results

**Figure 1:**
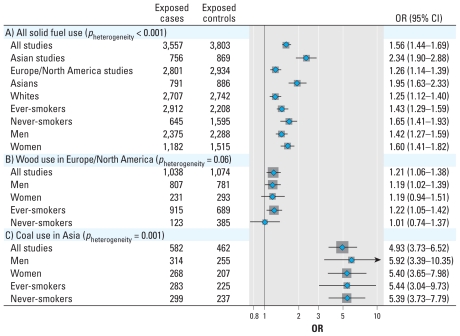
Lung cancer risk (ORs and 95% CIs) in all solid-fuel users (A), predominant wood users in North America and Europe (B), and predominant coal users in Asia (C) by sex and smoking status compared with subjects who used nonsolid fuels (gas, oil, electric), adjusted for age, sex, education, race/ethnicity, and smoking status. The gray squares represent the adjusted ORs, with the size proportional to the number of cases in that subgroup of analyses (i.e., overall, wood in Europe/North America, coal in Asia), and the horizontal lines represent the 95% CIs. *p*-Value for heterogeneity measures heterogeneity between studies. (A) All solid-fuel users (*n* = 1,548 unexposed cases and 2,732 unexposed controls). *p*-Value for heterogeneity between subgroups: men versus women, 0.18; ever- versus never-smokers, 0.14; whites versus Asians, 6.5 × 10^−5^; studies carried out in Asian versus North American and European countries, 1.9 × 10^−7^. (B) Predominant wood users in North America and Europe (*n* = 3,146 unexposed cases and 4,081 unexposed controls): includes only studies from North America and Europe (CEE, California, New England, Toronto), because the number of exposed cases from Asian countries was small (*n* = 94). The risk of lung cancer associated with wood use among all seven studies was 1.24 (95% CI, 1.10–1.41). Models also include interaction terms between the covariates and study. (C) Predominant coal users in Asia (*n* = 165 unexposed cases and 511 unexposed controls): includes only studies from Asia (Singapore, Xuanwei1, Xuanwei2). The risk of lung cancer associated with coal use was 1.64 (95% CI, 1.49–1.81) among all seven studies and 1.15 (95% CI, 1.02–1.30) among the four studies in North America and Europe.

We found that the cases tended to be older, more educated, and more likely to have smoked more than did the controls (Table 2). Predominant solid-fuel users had an increased risk of lung cancer compared with nonsolid-fuel users (Figure 1). We saw this increased risk among both men and women, in ever- and never-smokers, among whites and Asians, and among studies carried out in Asian and in North American and European countries.

Given the substantial heterogeneity for the risk of solid-fuel use across continents, we then examined coal and wood use separately and by studies carried out in Asian and North American and European countries. When we compared only predominant coal users and nonsolid-fuel users, we observed an increased risk of lung cancer (Figure 1). We saw this increased risk in studies in North America and Europe and particularly in studies in Asia. Further stratification showed that predominant coal users in Asia had an increased risk of lung cancer among men, women, and ever-smokers.

Similarly, predominant wood users had an increased risk of lung cancer compared with nonsolid-fuel users (Figure 1). This association was largely from studies conducted in North American and European countries because the number of exposed cases from Asian countries was small. Further stratification showed that predominant wood users from North American and European countries had an increased risk of lung cancer among men and never-smokers.

Nonsmoking women are of special interest because of their likely high exposure during household work such as cooking and because their results would likely suffer minimal residual confounding by tobacco use. Lung cancer was associated with coal use among never-smoking Asian women (OR = 5.41; 95% CI, 3.65–8.00); however, results for wood use among never-smoking Western women were more ambiguous (OR = 1.15; 95% CI, 0.81–1.64).

When restricting the analyses to only lifetime solid-fuel users (OR = 2.07; 95% CI, 1.80–2.38), lifetime coal users in Asia (OR = 2.85; 95% CI, 1.80–4.51), and lifetime wood users in North American and European countries (OR = 1.43; 95% CI, 0.97–2.11), the results were similar to those based on predominant use. Further, sensitivity analyses found lung cancer to be associated with solid-fuel use regardless of which study we excluded (CEE excluded: OR = 1.54; 95% CI, 1.37–1.73; California excluded: OR = 1.47; 95% CI, 1.34–1.62; Toronto excluded: OR = 1.57; 95% CI, 1.43–1.73; Singapore excluded: OR = 1.62; 95% CI, 1.48–1.77; Xuanwei1 excluded: OR = 1.23; 95% CI, 1.15–1.38; New England excluded: OR = 1.64; 95% CI, 1.50–1.79; Xuanwei2 excluded: OR = 1.55; 95% CI, 1.42–1.69).

For the studies that used population-based controls, lung cancer was associated with solid-fuel use (OR = 2.02; 95% CI, 1.72–2.38) and coal use in Asia (OR = 6.42; 95% CI, 4.24–9.72) but not wood use in North America and Europe (OR = 1.05; 95% CI, 0.78–1.40). Among studies using hospital-based controls, results were similar to the overall findings for the associations with solid-fuel use (OR = 1.12; 95% CI, 0.99–1.28) and wood use in North America and Europe (OR = 1.24; 95% CI, 1.05–1.46). The one study in Asia that used hospital-based controls did not have any cases or controls who were predominant coal users.

We assessed study heterogeneity for the association between lung cancer risk and fuel use. We observed study heterogeneity among predominant coal users (*p*_heterogeneity_ = 0.001), mainly attributed to the strong association observed in Xuanwei1. When we excluded Xuanwei1 from the analysis, residual heterogeneity was within that expected from random variation (*p*_heterogeneity_ = 0.31), and the association between coal use and lung cancer risk remained. Study heterogeneity for wood use in Western countries was also within that expected from random variation (*p*_heterogeneity_ = 0.06).

## Discussion

We pooled seven studies from North America, Europe, and Asia to evaluate solid-fuel use and lung cancer risk and found an association between lung cancer and coal use in Asia, which is consistent with previous studies. Wood use in North American and European countries was also associated with lung cancer risk in our analysis. These associations persisted when we stratified by demographic characteristics.

Our observed association between coal use and lung cancer risk is consistent with previous case–control studies (Galeone et al. 2008; Lan et al. 2000; Xu et al. 1989) and cohort studies (Hosgood et al. 2008; Lan et al. 2002). These results are unsurprising because coal combustion products are known to contain carcinogens such as PAHs (IARC 1983; Zhang and Smith 2003), and exposure to in-home coal combustion smoke is a classified lung carcinogen (IARC 2010). The association between coal use and lung cancer risk among never-smoking Asian women supports the idea that in-home coal smoke is a lung cancer risk factor that is independent of smoking. Further, after excluding each of the studies conducted in Xuanwei, coal use remained associated with lung cancer risk, suggesting that the carcinogenic potential of coal is not restricted to a single geographic area.

Wood smoke has been associated with respiratory diseases, such as chronic obstructive pulmonary disease (Ezzati and World Health Organization 2004; Orozco-Levi et al. 2006). Although a few studies have observed suggestive associations between lung cancer risk and in-home wood use (Behera and Balamugesh 2005; Hernández-Garduño et al. 2004; Lissowska et al. 2005; Pisani et al. 2006), other studies have not replicated these findings (Gao et al. 1987; Sapkota et al. 2008). Our results are consistent with an association between wood use and lung cancer among women, which seems likely because they tend to spend more time at home and thus have greater exposures to solid-fuel combustion products than do men. The association of wood use and lung cancer risk observed in our analysis is important, because IARC classified biomass use (primarily wood) as a group 2A carcinogen due to limited epidemiological evidence (IARC 2010).

To the extent allowed by measurement error, we were able to control for some important confounders, such as smoking, age, and education. Our large sample size also enabled us to explore heterogeneity by race/ethnicity, sex, geographic location, and smoking status. Nonetheless, the inclusion of additional studies would improve some of our subgroup analyses, such as wood users in Asia.

A limitation of our study is that some studies used hospital-based controls, whereas others used population-based controls. Control selection, however, is unlikely to explain our key findings, because solid-fuel use and coal use in Asia were associated with lung cancer when restricted to studies with population-based controls. On the other hand, wood use in Western countries was not associated with lung cancer among the two studies with population-based controls, which were carried out in the United States, but was associated with lung cancer in the substantially larger hospital-based CEE Study, which was carried out in Central and Eastern Europe. It is unknown whether this difference is driven by control type, by other differences such as the lower prevalence of exposure among controls in the North American studies, or by other factors related to the potential dose experienced by the subjects. An additional limitation is that fuel use exposure assessments were questionnaire-based self-reports without quantitative environmental measurements, so there is potential for differential misclassification. Furthermore, questionnaires varied across studies, so the degree of misclassification bias also may have varied.

Another limitation of the present analysis is that we were able to assess only the type of fuel used. Information on intensity and duration of fuel use, time spent indoors, the type of stove used, and quality of ventilation in the home would refine our study, because these factors have been shown to influence the lung cancer risk associated with solid-fuel use (Hosgood et al. 2008; Lan et al. 2002). These factors are particularly important when comparing Asian and Western countries, such as Europe and North America, because the indoor exposures may vary. We have developed a standardized questionnaire including this information for ongoing and future studies, which would increase comparability of results and aid in data pooling. The inclusion of qualitative exposure assessment methods to better estimate the amount of fuel used throughout the subjects’ lives, and the integration of quantitative exposure assessment methodologies to measure the subjects’ doses are crucial to answer open research questions, such as dose–response relationships.

We could not adjust for other indoor sources of lung carcinogens, such as environmental tobacco smoke (ETS). However, given the size of our observed associations and given that ETS is associated with only about a 20% increased risk of lung cancer (IARC 2004; Taylor et al. 2007; Zhong et al. 2000), and because the association between solid-fuel use and lung cancer was not attenuated after adjusting for ETS in one study in this analysis (Lan et al. 2008), we believe it is unlikely that confounding by ETS could fully explain our results. Finally, radon exposure may confound our results, because some geographic locations and household characteristics, such as underground dwellings and ventilation factors, have been associated with lung cancer (Lubin et al. 2004). However, because none of our study populations systematically resided in underground dwellings and because indoor radon levels were at or below background levels when measured (Xuanwei, China), we think it is unlikely that residual confounding from radon exposure could explain our findings completely.

## Conclusion

Our pooled-analysis of 11,640 individuals from three continents confirms the association between coal use and lung cancer risk and provides epidemiological evidence that wood users are at an increased risk of lung cancer. Further research is necessary to elucidate the potential modification of these associations by genetic variation (Hosgood et al. 2007), varying carcinogenic potential among particular fuel subtypes (Lan et al. 2008), and varying carcinogenic potential by stove type and dwelling characteristics.

## Figures and Tables

**Table 1:** Summary of case–control studies pooled for indoor air pollution analysis.^a^

Study sponsor (study name)	Principal investigator	Country of study	Study period	Control source	Cases	Controls	Participation rate (%)	
							Cases	Controls
North American and European studies								

Central and Eastern Europe (CEE)	P. Boffetta	Central and Eastern Europe	1998–2002	Hospital based	2,861	2,936	84	85
University of California–Los Angeles (California)	Z.F. Zhang	United States	1999–2004	Population based	611	1,040	39	72
New England Lung Cancer Study (New England)	E. Duell	United States	2005–2008	Population based	277	251	61	46
Samuel Lunenfeld Research Institute (Toronto)	J. McLaughlin	Canada	1997–2002	Population and hospital based	445	962	62	71

Asian studies								

National University of Singapore (Singapore)	A. Seow	Singapore	1996–1998	Hospital based	303	763	95	97
National Cancer Institute (Xuanwei1)	Q. Lan	China	1985–1990	Population based	498	498	100	97
National Cancer Institute (Xuanwei2)	Q. Lan	China	1995–1996	Population based	122	122	98	100

a As previously reported by Hashibe et al. (2006; California), Heck et al. (2009; New England), Hung et al. (2008; Toronto), Lan et al. (2000; Xuanwei2), Lan et al. (2008; Xuanwei1), Scélo et al. (2004; CEE), and Seow et al. (2000; Singapore).

**Table 2:** Characteristics of pooled indoor air pollution study population.

	Cases (*n* = 5,105)		Controls (*n* = 6,535)		
Characteristic	*n*	%	*n*	%	*p*-Value^a^
Sex					< 0.0001

Men	3,176	62.2	3,600	55.1	
Women	1,926	37.7	2,935	44.9	

Age (years)					< 0.0001

< 50	875	17.1	1,553	23.8	
50–60	1,878	36.8	2,330	35.7	
> 60–70	1,584	31.0	1,664	25.5	
> 70	768	15.0	998	15.3	

Race/ethnicity					< 0.0001

White	3,825	74.9	4,412	67.5	
Asian	1,059	20.7	1,544	23.6	
Other	221	4.3	579	8.9	

Education					< 0.0001

Low (0–9 years)	1,302	25.5	1,871	28.6	
Medium (10–15 years)	2,795	54.8	2,981	45.6	
High (≥16 years)	1,008	19.7	1,683	25.8	

Smoking status					< 0.0001

Ever	4,116	80.6	3,524	53.9	
Never	989	19.4	3,011	46.1	

Geographic region					< 0.0001

North America	1,329	26.0	2,226	34.1	
Asia	921	18.0	1,380	21.1	
Europe	2,855	55.9	2,929	44.8	

Fuel type predominantly used					< 0.0001^b^

Nonsolid fuels (electric, oil, gas)	1,548	30.3	2,732	41.8	
Solid fuels (coal, wood)	3,557	69.7	3,803	58.2	
Coal only	1,943	38.1	1,945	29.8	
Wood only	1,080	21.2	1,172	17.9	

a Chi-square test.

b Comparing nonsolid-fuel users and solid-fuel users.
